# Prioritizing the Role of Major Lipoproteins and Subfractions as Risk Factors for Peripheral Artery Disease

**DOI:** 10.1161/CIRCULATIONAHA.121.053797

**Published:** 2021-06-18

**Authors:** Michael G. Levin, Verena Zuber, Venexia M. Walker, Derek Klarin, Julie Lynch, Rainer Malik, Aaron W. Aday, Leonardo Bottolo, Aruna D. Pradhan, Martin Dichgans, Kyong-Mi Chang, Daniel J. Rader, Philip S. Tsao, Benjamin F. Voight, Dipender Gill, Stephen Burgess, Scott M. Damrauer

**Affiliations:** 1 Division of Cardiovascular Medicine (M.G.L.), University of Pennsylvania Perelman School of Medicine, Philadelphia.; 2 Department of Medicine (M.G.L., K.-M.C., D.J.R.), University of Pennsylvania Perelman School of Medicine, Philadelphia.; 3 Department of Surgery (V.M.W., S.M.D.), University of Pennsylvania Perelman School of Medicine, Philadelphia.; 4 Institute for Translational Medicine and Therapeutics (D.J.R., B.F.V.), University of Pennsylvania Perelman School of Medicine, Philadelphia.; 5 Department of Genetics (D.J.R., B.V.F.), University of Pennsylvania Perelman School of Medicine, Philadelphia.; 6 Department of Systems Pharmacology and Translational Therapeutics (B.V.F.), University of Pennsylvania Perelman School of Medicine, Philadelphia.; 7 Corporal Michael J. Crescenz VA Medical Center, Philadelphia, PA (M.G.L., K.-M.C., B.F.V., S.M.D.).; 8 MRC Biostatistics Unit (V.Z., S.B.), School of Clinical Medicine, University of Cambridge, UK.; 9 Department of Medical Genetics (L.B.), School of Clinical Medicine, University of Cambridge, UK.; 10 BHF Cardiovascular Epidemiology Unit (S.B.), School of Clinical Medicine, University of Cambridge, UK.; 11 Department of Epidemiology and Biostatistics (V.Z.), Imperial College London, UK.; 12 Dementia Research Institute (V.Z.), Imperial College London, UK.; 13 Department of Epidemiology and Biostatistics (D.G.), Imperial College London, UK.; 14 Medical Research Council Integrative Epidemiology Unit, University of Bristol, UK (V.M.W.).; 15 Malcolm Randall VA Medical Center, Gainesville, FL (D.K.).; 16 Department of Surgery, University of Florida, Gainesville (D.K.).; 17 VA Informatics and Computing Infrastructure, Department of Veterans Affairs, Salt Lake City Health Care System, UT (J.L.).; 18 University of Utah School of Medicine, Salt Lake City (J.L.).; 19 Institute for Stroke and Dementia Research, University Hospital of Ludwig-Maximilians-University, Munich, Germany (R.M.).; 20 Vanderbilt Translational and Clinical Cardiovascular Research Center, Division of Cardiovascular Medicine, Vanderbilt University Medical Center, Nashville, TN (A.W.A.).; 21 The Alan Turing Institute, London, UK (L.B.).; 22 Division of Preventive Medicine, Brigham and Women’s Hospital, Harvard Medical School, Boston, MA (A.D.P.).; 23 Division of Cardiovascular Medicine, VA Boston Medical Center, MA (A.D.P.).; 24 Institute for Stroke and Dementia Research, University Hospital of Ludwig-Maximilians-University, Munich, Germany (M.D.).; 25 German Center for Neurodegenerative Diseases (DZNE), Munich, Germany (M.D.).; 26 Munich Cluster for Systems Neurology (SyNergy), Germany (D.J.R., B.F.V.).; 27 Palo Alto VA Healthcare System, CA (P.S.T.).; 28 Department of Medicine, Division of Cardiovascular Medicine, and Stanford Cardiovascular Institute, Stanford University, Palo Alto, CA (P.S.T.).; 29 Clinical Pharmacology and Therapeutics Section, Institute for Infection and Immunity, St. George’s, University of London, UK (D.G.).; 30 Novo Nordisk Research Centre Oxford, Old Road Campus, UK (D.G.).

**Keywords:** atherosclerosis, coronary artery disease, genomics, lipoproteins, peripheral arterial disease

## Abstract

Lipoprotein-related traits have been consistently identified as risk factors for atherosclerotic cardiovascular disease, largely on the basis of studies of coronary artery disease (CAD). The relative contributions of specific lipoproteins to the risk of peripheral artery disease (PAD) have not been well defined. We leveraged large-scale genetic association data to investigate the effects of circulating lipoprotein-related traits on PAD risk.

**Methods:**

Genome-wide association study summary statistics for circulating lipoprotein-related traits were used in the mendelian randomization bayesian model averaging framework to prioritize the most likely causal major lipoprotein and subfraction risk factors for PAD and CAD. Mendelian randomization was used to estimate the effect of apolipoprotein B (ApoB) lowering on PAD risk using gene regions proxying lipid-lowering drug targets. Genes relevant to prioritized lipoprotein subfractions were identified with transcriptome-wide association studies.

**Results:**

ApoB was identified as the most likely causal lipoprotein-related risk factor for both PAD (marginal inclusion probability, 0.86; *P*=0.003) and CAD (marginal inclusion probability, 0.92; *P*=0.005). Genetic proxies for ApoB-lowering medications were associated with reduced risk of both PAD (odds ratio,0.87 per 1-SD decrease in ApoB [95% CI, 0.84–0.91]; *P*=9×10^−10^) and CAD (odds ratio,0.66 [95% CI, 0.63–0.69]; *P*=4×10^−73^), with a stronger predicted effect of ApoB lowering on CAD (ratio of effects, 3.09 [95% CI, 2.29–4.60]; *P*<1×10^−6^). Extra-small very-low-density lipoprotein particle concentration was identified as the most likely subfraction associated with PAD risk (marginal inclusion probability, 0.91; *P*=2.3×10^−4^), whereas large low-density lipoprotein particle concentration was the most likely subfraction associated with CAD risk (marginal inclusion probability, 0.95; *P*=0.011). Genes associated with extra-small very-low-density lipoprotein particle and large low-density lipoprotein particle concentration included canonical ApoB pathway components, although gene-specific effects were variable. Lipoprotein(a) was associated with increased risk of PAD independently of ApoB (odds ratio, 1.04 [95% CI, 1.03–1.04]; *P*=1.0×10^−33^).

**Conclusions:**

ApoB was prioritized as the major lipoprotein fraction causally responsible for both PAD and CAD risk. However, ApoB-lowering drug targets and ApoB-containing lipoprotein subfractions had diverse associations with atherosclerotic cardiovascular disease, and distinct subfraction-associated genes suggest possible differences in the role of lipoproteins in the pathogenesis of PAD and CAD.

## Abstract 2

Supplemental Digital Content is available in the text.

**Clinical Perspective**

**What Is New?**

- Apolipoprotein B was identified as the most likely casual lipoprotein-related risk factor for peripheral artery disease (PAD), and apolipoprotein B (ApoB)–lowering medications were predicted to reduce the risk of PAD.
- The effect of ApoB (and ApoB-lowering medications) was predicted to be ≈3 times greater for coronary artery disease than PAD.
- Extra-small very-low-density lipoprotein was prioritized as the most likely ApoB-containing subfraction associated with PAD risk, whereas large low-density lipoprotein particle concentration was prioritized as the most likely subfraction associated with coronary artery disease.

**What Are the Clinical Implications?**

- Interventions targeting ApoB are likely to reduce the risk of PAD.
- Future studies may be warranted to test whether interventions targeting specific lipoprotein-related subfractions may reduce the risk of PAD and coronary artery disease.

Atherosclerotic cardiovascular disease (ASCVD) is the most common cause of morbidity and mortality worldwide.^1^ Most research has focused on ASCVD in the coronary arteries (coronary artery disease [CAD]). However, peripheral artery disease (PAD) represents another common and often underrecognized manifestation of ASCVD that is also associated with significant morbidity (eg, pain, tissue loss, amputation) and mortality, affecting >5% of the global adult population.^2,3^ Dyslipidemia has been a long-established risk factor for ASCVD, with the strongest evidence derived from large studies focused primarily on CAD end points. Although low-density lipoprotein (LDL) cholesterol (LDL-C)–reducing medications such as statins are commonly used in the prevention and treatment of PAD, evidence for the relationship between LDL-C and PAD risk has been inconsistent.^4^ Observational studies with modest event rates have suggested that components of the atherogenic dyslipidemia profile (elevated levels of triglyceride-rich lipoproteins, small LDL-C, and the ratio of total cholesterol to high-density lipoprotein cholesterol [HDL-C], along with low concentrations of HDL-C) may be more strongly associated with PAD than CAD, although the relative contribution of the major circulating lipoproteins (LDL-C, HDL-C, triglycerides, apolipoprotein B [ApoB], and apolipoprotein A1) and associated lipoprotein subfractions to PAD specifically has remained poorly defined.^4–7^

Over the past 15 years, genome-wide association studies (GWASs) have identified hundreds of genetic loci associated with ASCVD traits, major lipoproteins, and related subfractions.^8–12^ The large data sets arising from these studies include genetic associations estimated in hundreds of thousands of participants. An array of analytical methods enable the analysis of these genetic data sets to provide insights into the underlying biology of diseases. Mendelian randomization (MR) uses genetic variants as instrumental variables to infer the effect of an exposure on an outcome, under the assumption that genetic associations with the outcome are mediated via the exposure for selected variants.^13^ MR has been used to implicate ApoB as an important risk factor for CAD and to validate the effects of drug targets on disease outcomes.^14–19^ Integrating genetic data with gene transcription data sets in transcriptome-wide association studies (TWASs) has been used to identify tissue-level gene expression associations with disease (such as between hepatic expression of *SORT1* and risk of CAD).^20^

We aimed to integrate large-scale genetic data sets (1) to prioritize the role of circulating lipoproteins and subfractions on PAD risk, (2) to identify genes that may represent novel lipoprotein-pathway targets in the prevention and treatment of PAD, and (3) to estimate the effects of current/potential lipid-lowering medications on PAD risk.

## Methods

### Data Availability

The data that support the findings of this study are available from the corresponding author on reasonable request. GWAS summary statistics for PAD are available by application in dbGaP (phs001672). GWAS summary statistics for Global *Lipids* Genetics Consortiumlipids, UK Biobank lipids, nuclear magnetic resonance (NMR) lipids, and CARDIoGRAMplusC4D CAD are available for download from the Integrative Epidemiology Unit Open GWAS Project.

### Ethics Approval

The Veterans Affairs Central Institutional Review Board approved the MVP (Million Veteran Program) study protocol.

### Study Population and Outcomes

Our primary outcome was PAD. Genetic associations with PAD were derived from a 2019 GWAS by Klarin et al.^10^ Full summary data are available by application to dbGaP (phs001672). This study included 31 307 PAD cases (24 009 European ancestry, 5373 African ancestry, 1925 Hispanic ancestry) and 211 753 controls among participants of the Veterans Affairs MVP, which recruited individuals 19 years of age from Veterans Affairs medical centers across the United States.^21^ PAD diagnoses were ascertained from electronic health records using *International Classification of Diseases* 9th and 10th revision and Current Procedural Terminology codes. Genetic associations were performed separately by ancestry groups with logistic regression adjusted for age, sex, and 5 ancestry-specific genetic principal components and then combined using an inverse-variance weighted fixed-effects method.

CAD was included as an outcome in our analysis to help contextualize the PAD results because most of the observational, MR, and randomized control trial data relating to ASCVD have focused on CAD outcomes. Genetic associations with CAD were derived from the CARDIoGRAMplusC4D 1000 Genomes GWAS.^11^ This is a meta-analysis of 48 studies, including 60 801 CAD cases and 123 504 controls of European ancestries (77%), including combination incident and prevalent CAD among the cases. CAD case/control status was determined at the individual study level, with CAD cases included on the basis of the presence of myocardial infarction, acute coronary syndrome, chronic stable angina, or coronary stenosis of >50%.

### Prioritizing the Role of Major Lipoprotein-Related Traits and Lipoprotein Subfractions on PAD

**Figure 1.**
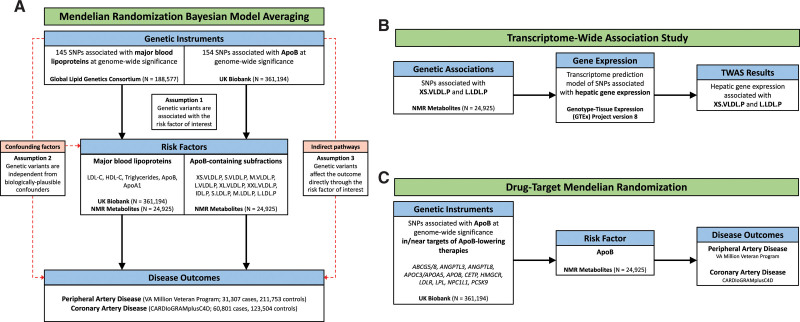
**Overview of risk factor prioritization, drug target, and transcriptome-wide association study (TWAS) analyses.** Overview of main analyses. **A**, Risk factor prioritization was performed with mendelian randomization (MR) bayesian model averaging to prioritize the contribution of major lipoproteins and apolipoprotein (Apo) B–containing subfractions to peripheral artery disease (PAD) and coronary artery disease (CAD) risk. The primary MR assumptions are denoted, with red dashed lines representing violations of the MR assumptions. **B**, A TWAS integrating gene expression and genetic association data was performed to identify putative genes involved in the regulation of the prioritized ApoB-containing subfractions. **C**, Drug target MR was performed to identify the effect of genes encoding targets of ApoB-lowering medications on PAD and CAD outcomes. FDR indicates false discovery rate; HDL-C, high-density lipoprotein cholesterol; IDL.P, intermediate-density lipoprotein particles; L.LDL.P, large large-density lipoprotein particles; L.VLDL.P, large very-large-density lipoprotein particles; M.LDL.P, medium large-density lipoprotein particles; M.VLDL.P, medium very-large-density lipoprotein particles; NMR, nuclear magnetic resonance; PAD, peripheral artery disease; S.LDL.P, small large-density lipoprotein particles; SNP, single nucleotide polymorphism; S.VLDL.P, small very-large-density lipoprotein particles; XL.VLDL.P, extralarge very-large-density lipoprotein particles; XS.VLDL.P, extra-small very-large-density lipoprotein particles; and XXL.VLDL.P, extra-extralarge very-large-density lipoprotein particles.

We performed 2 MR bayesian model averaging (MR-BMA)^14^ analyses as illustrated in Figure 1A. MR-BMA is an extension of multivariable MR that applies a bayesian variable selection method and aims to identify true causal risk factors (rather than the magnitude of effect) by jointly considering correlated exposures (in this case, lipoprotein-related traits).^14,22,23^ Details of the MR-BMA method are available in Methods in the Data Supplement. First, to identify causal relationships between major lipoprotein-related traits and PAD, our exposures of interest were circulating lipoproteins (LDL-C, HDL-C), their primary constituent apolipoproteins (apolipoprotein A1 and ApoB), and triglycerides (Figure 1A). Second, to investigate whether the predicted effect of ApoB lowering on PAD may be influenced by specific lipoprotein subfractions, we performed a further MR-BMA analysis focusing on 10 ApoB-containing lipoprotein subfractions (Figure 1A). For comparison, we also performed these analyses for CAD.

The instrumental variables consisted of 145 independent (*r*^2^<0.001 in the 1000 Genomes European-ancestry Reference Panel) genetic variants associated with any major lipoprotein-related trait (total cholesterol, LDL-C, HDL-C, or triglycerides) at a genome-wide significance level (*P*<5×10^−8^) in the Global Lipids Genetics Consortium GWAS, an analysis comprising 60 individual studies of primarily European-ancestry participants.^8^ Genetic associations with circulating levels of major lipoprotein-related traits in blood were estimated in the UK Biobank study based on 361 194 European-ancestry participants. Genetic associations were adjusted for age, sex, and 20 principal components. Genetic associations with lipoprotein subfractions were estimated from a GWAS of circulating lipoproteins and subfractions in 24 925 European-descent participants.^9^ In this data set, lipoproteins and subfractions were measured with NMR spectroscopy. Genetic associations were adjusted for age, sex, and the first 10 genetic principal components. As a replication analysis, we repeated the analysis for major lipoprotein-related traits using genetic associations from the NMR data set.

### Transcriptome-Wide Association Study

After prioritizing the role of lipoprotein subfractions in PAD, we next sought to identify genes associated with those subfractions, which may ultimately serve as therapeutic targets (Figure 1B). To identify genes associated with circulating levels of lipoprotein subfractions, TWASs were performed using S-PrediXcan.^20^ This tool enables the integration of tissue-level expression quantitative trait loci data sets with GWAS summary statistics to prioritize genes associated with traits of interest. Because the liver plays a critical role in lipoprotein metabolism, we obtained a pretrained transcriptome prediction model for liver gene expression derived from the Genotype-Tissue Expression Project version 8. Predicted liver gene expression and GWAS summary statistics for lipoprotein subfractions were then correlated using S-PrediXcan to identify genes significantly associated with circulating levels of lipoprotein subfractions. The significance of differences in the effect of each gene on each outcome was determined by 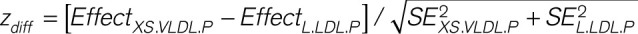 with *P* values derived from the normal distribution. We performed gene ontology enrichment analysis using ShinyGO to identify Gene Ontology Biological Processes significantly associated with the genes prioritized by S-PrediXcan.^24^ Finally, we performed a combined multivariate and collapsing test to investigate the effect of rare damaging mutations in genes prioritized by the TWAS on risk of PAD among participants of the UK Biobank who underwent whole-exome sequencing (Methods in the Data Supplement).^25^

### Genetically Proxied ApoB Lowering and PAD Risk

To predict the impact of ApoB lowering on PAD and CAD risk, we performed further MR analyses (Figure 1C). We performed gene-based analyses using variants associated with ApoB in gene regions that proxy specific lipid-lowering drugs (licensed or proposed) and polygenic analyses using all such variants. Genetic variants associated with ApoB levels at genome-wide significance (*P*<5×10^−8^) were identified from the UK Biobank and pruned at *r*^2^<0.1 to exclude highly correlated variants. This set of variants was further narrowed into 2 biologically informed sets. First, we examined variants located in or near (±200 kb) genes encoding previously identified regulators of ApoB metabolism (*ABCG5/8*, *ANGPTL3*, *ANGPTL4*, *ANGPTL8*, *APOC3/APOA5*, *APOB*, *CETP*, *DGAT*, *HMGCR*, *LDLR*, *LPL*, *MTTP*, *NPC1L1*, *PCSK9*, and *PPARA*), representing the targets of current or proposed therapeutics.^16^ Next, we examined variants in or near (±200 kb) genes associated with extra-small very-low-density lipoprotein (VLDL) particle concentration (XS.VLDL.P) in the TWAS analysis (false discovery rate [FDR] *q*<0.05).^26^ Genetic associations with ApoB were taken from the NMR data set to avoid winner’s curse and sample overlap.^9^ MR estimates were obtained from the random-effects inverse variance–weighted method performed using the MendelianRandomization package in R, accounting for linkage disequilibrium correlation among variants using the 1000 Genomes Phase 3 European reference panel. The MR-Egger method, which makes different assumptions about the presence of pleiotropy at the cost of decreased statistical power, was performed as a sensitivity analysis when >2 single nucleotide polymorphisms were present in the genetic instrument.^27^

### Lipoprotein(a) and PAD Risk

To examine the relationship between lipoprotein(a) Lp(a) and PAD, we performed 2-sample MR using summary statistics. As genetic instruments for Lp(a), we used 15 common genetic variants that are conditionally independent predictors of Lp(a), had previously been shown to explain >40% of the variance in circulating Lp(a) levels, and were present in GWAS summary statistics for both PAD and CAD. We obtained genetic association effect estimates from a previous analysis.^28^ We performed inverse variance–weighted MR, considering PAD as the primary outcome, with CAD presented for comparison. We also performed multivariable MR, accounting for the associations between these genetic variants and ApoB in the UK Biobank biomarker GWAS.

### Statistical Analysis

For the main MR-BMA analyses of major lipoprotein-related traits and PAD, FDR correction was performed to account for multiple testing, with FDR-corrected *q*<0.05 set as the predetermined significance threshold.^15^ For the MR-BMA analysis of lipoprotein subfractions, the Nyholt procedure of effective tests was used to account for the strong correlation among the subfractions, with a multiple testing–adjusted value of *P*=0.05 set as the significance threshold.^29^ For the drug target MR analysis, *P*<0.05 was the predetermined significance threshold. For the TWAS and gene ontology enrichment analyses, the FDR was used to account for multiple testing, with FDR-corrected *P*<0.05 set as the predetermined significance threshold. To compare the influence of lipoprotein risk factors on PAD versus CAD, we calculated the ratio of effects (log-odds), with 95% CIs obtained by bootstrap resampling. All statistical analyses were performed with R version 4.0.3 (R Foundation for Statistical Computing). This study is reported in accordance with the Strengthening the Reporting of Observational Studies in Epidemiology guidelines for reporting observational studies.^30^

## Results

### Prioritizing the Role of Major Lipoprotein-Related Traits and Lipoprotein Subfractions on PAD

In the MR-BMA analysis for major lipoprotein-related traits, ApoB was the top-ranked risk factor for PAD (marginal inclusion probability, 0.86; *P*=0.003; Table 1 and Tables I–III in the Data Supplement). In the replication analysis, in which genetic association estimates for the 5 major lipoprotein-related traits were derived from the NMR metabolite GWAS, ApoB was again the top-ranked risk factor for PAD with a marginal inclusion probability of 0.68 (*P*=0.001; Tables IV–VI in the Data Supplement). Similarly, ApoB was identified as the prioritized risk factor for CAD in the primary (marginal inclusion probability, 0.92; *P*=0.005; Table 1) and replication (marginal inclusion probability, 0.80; *P*=0.004) analyses, in keeping with the previously established role of ApoB in CAD (Tables VII and VIII in the Data Supplement).^15,16^ These results provide strong, consistent support for the role of ApoB as the primary lipoprotein risk factor for PAD and CAD.

**Table 1.**
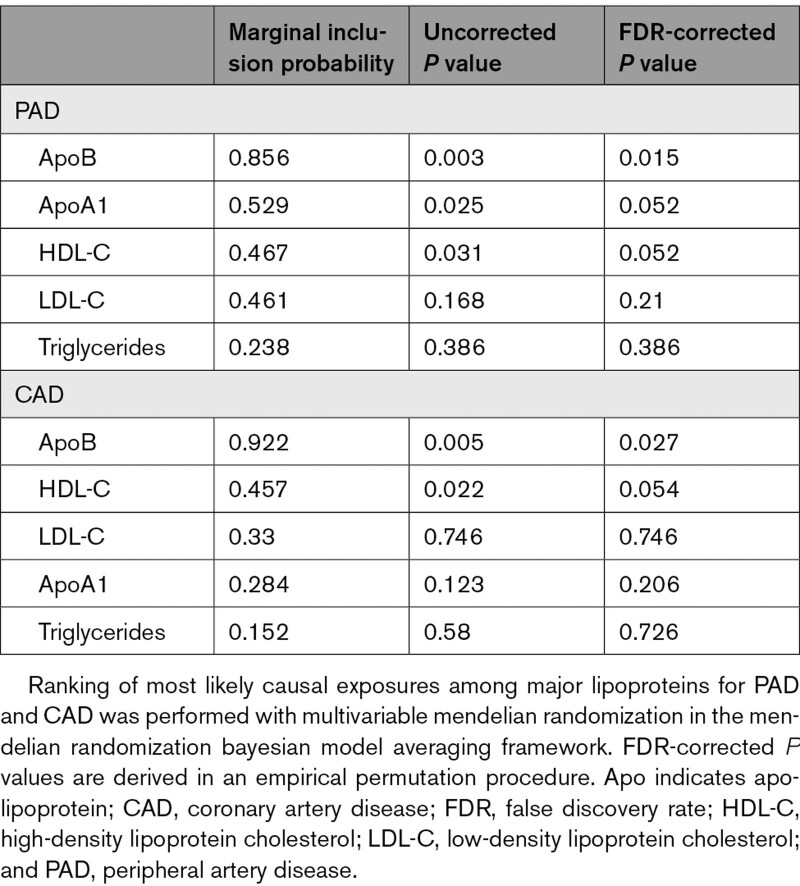
Prioritization of Causal Risk Factors Among Major Lipoproteins

In the MR-BMA analysis for lipoprotein subfractions, XS.VLDL.P was prioritized as the primary ApoB-containing risk factor for PAD (marginal inclusion probability, 0.91; *P*=2.3×10^−4^; Table 2 and Table IX in the Data Supplement). In contrast, large LDL particle concentration (L.LDL.P) was prioritized as the primary ApoB-containing risk factor for CAD (marginal inclusion probability, 0.95; *P*=0.011; Table 2 and Table X in the Data Supplement).

**Table 2.**
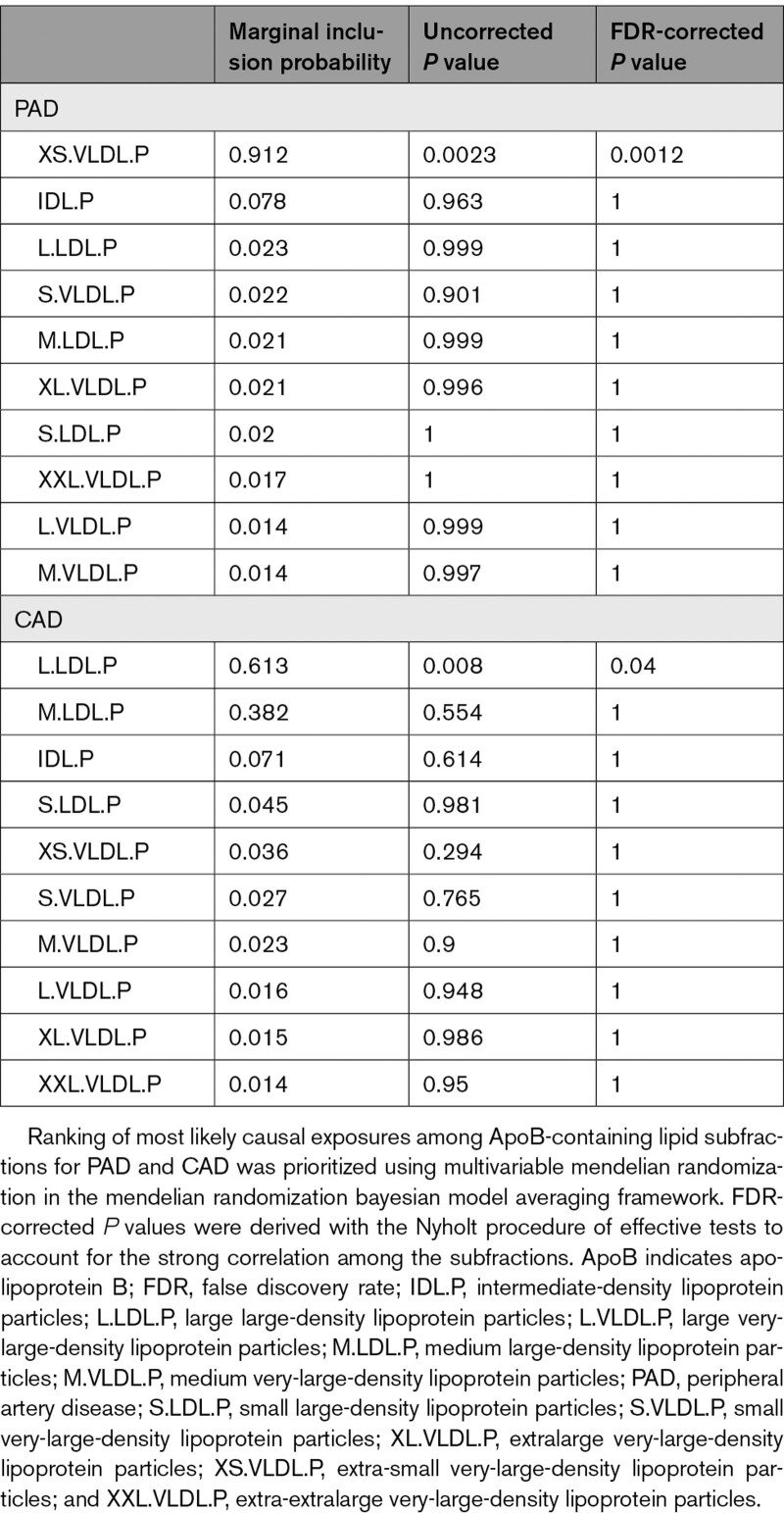
Prioritization of Causal Risk Factors Among ApoB-Containing Lipid Subfractions

### Identification of Genes Associated With ApoB-Containing Lipoprotein Subfractions

Having prioritized XS.VLDL.P and L.LDL.P as important ApoB-containing lipoprotein subfractions for PAD and CAD, respectively, we explored whether specific genes may influence the circulating levels of these subfractions. Given the key role of the liver in lipoprotein metabolism, we integrated hepatic gene expression data from the Genotype-Tissue Expression Project with the XS.VLDL.P and L.LDL.P GWAS summary statistics to identify genes associated with circulating levels of each ApoB-containing lipoprotein subfraction.

**Figure 2.**
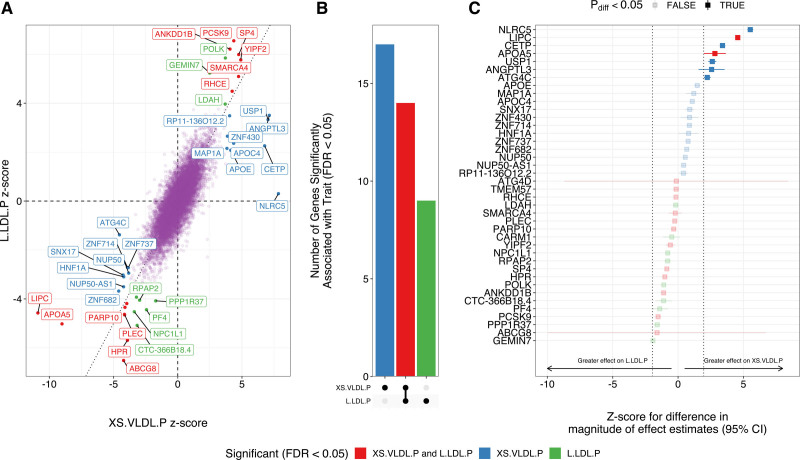
**Genes associated with circulating levels of extra-small very-low-density lipoprotein particle concentration (XS.VLDL.P) and large low-density lipoprotein particle concentration (L.LDL.P) lipoprotein subfractions.** Transcriptome-wide association studies were performed integrating liver gene expression data from Genotype-Tissue Expression version 8 with genome-wide association study (GWAS) summary statistics for XS.VLDL.P and L.LDL.P to identify genes associated with circulating levels of each lipoprotein subfraction. **A**, Genes significantly (false discovery rate [FDR] <0.05) associated with either subfraction are labeled, and colors represent the subfraction associations. **B**, Bar plot depicts the number of unique and shared genes between the 2 subfractions. **C**, Forest plot depicts the *z* score for the difference in the effect magnitude for each gene on each subfraction (|β_XS.VLDL.P_|−|β_L.LDL.P_|). Dotted lines represent *z* scores of ±1.96, with point estimates outside this range representing significant (*P*_diff_<0.05) differential effects. Error bars represent 95% CIs for the *z* score.

Using TWAS, we identified 31 genes associated with XS.VLDL.P and 23 genes associated with L.LDL.P, for a total of 40 unique genes (FDR <0.05 for either subfraction; Tables XI and XII in the Data Supplement). Of these 40 genes, 17 were uniquely associated with XS.VLDL.P levels, 9 were uniquely associated with L.LDL.P levels, and 14 were associated with circulating levels of both subfractions (Figure 2A and 2B). As expected, genes associated with these lipoprotein subfractions were significantly enriched for membership in cholesterol metabolism and related pathways (Table XIII in the Data Supplement). Among the genes associated with both subfractions were several canonical genes involved in lipoprotein metabolism, including *PCSK9*, *ABCG8*, *LIPC*, and *APOA5*. The 17 genes uniquely associated with XS.LDL.P levels were enriched for clusters of biological processes involving triglyceride-rich lipoprotein metabolism (Table XIV in the Data Supplement), whereas the 9 genes uniquely associated with L.LDL.P levels were not significantly enriched for specific biological processes.

Across the genome, there was overall a strong correlation between the estimated effects of gene expression on circulating XS.VLDL.P and L.LDL.P levels (Pearson correlation=0.80, *P*<2.2×10^−16^). However, among the 40 genes significantly associated with either lipoprotein subfraction, we identified potentially heterogeneous effects. For example, *NLRC5*, *LIPC*, *CETP*, *APOA5*, *USP1*, *ANGPTL3*, and *ATGC4* expression was predicted to more strongly affect circulating XS.VLDL.P levels compared with L.LDL.P (Figure 2C).

To investigate the impact of these genes on PAD risk, we examined whether damaging mutations in XS.VLDL.P–associated genes might influence PAD risk among UK Biobank participants. We identified rare loss-of-function variants in 29 of 31 XS.VLDL.P–associated genes among 154 584 UK Biobank participants (1668 PAD cases and 152 916 controls). After multiple testing was accounted for, only damaging variants in *SP4* were associated with prevalent PAD (FDR *q*=0.049; Table XV in the Data Supplement).

### Genetically Predicted ApoB Lowering and PAD Risk

**Figure 3.**
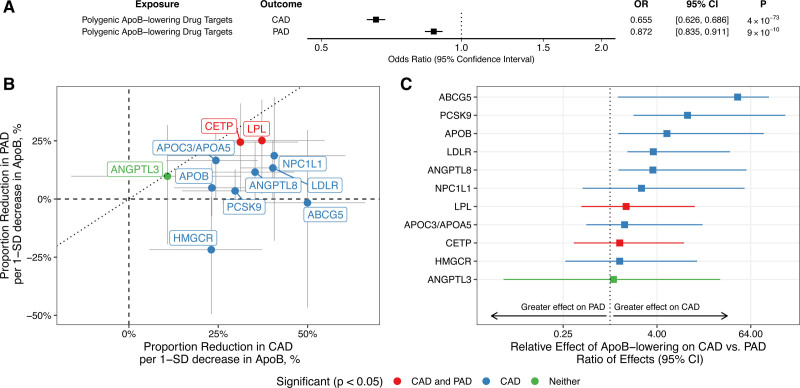
**Associations between genetically predicted apolipoprotein B (ApoB) levels and peripheral artery disease (PAD).** Estimates represent associations between genetically proxied ApoB and PAD or coronary artery disease (CAD) risk, scaled to the change in disease risk per 1-SD decrease in ApoB. Genetic variants used to proxy reductions in ApoB included only those located near of within genes important in ApoB metabolism. **A**, Polygenic analysis including all such variants. **B**, Gene-based analyses considering variants for each gene region. The dotted line with slope of 1 represents the scenario in which the association of genetically proxied ApoB with disease risk is equal for both CAD and PAD. **C**, Relative effects of each gene region on CAD vs PAD as determined by the ratio of effects. Error bars represent 95% CIs. OR indicates odds ratio.

Because MR has previously been used to predict the impact of current and proposed ApoB-lowering therapies on CAD risk, we sought to explore the effect of these treatments on PAD risk.^16^ We first performed polygenic and gene-based MR analyses to determine whether ApoB-associated genetic variants located within/near genes encoding these therapeutic targets were associated with risk of PAD. In polygenic analyses, genetically proxied ApoB lowering was associated with reduced risk of PAD (odds ratio [OR], 0.87 per 1-SD reduction in ApoB [95% CI, 0.84–0.91]; *P*=9×10^−9^; Figure 3A). As a comparison, the association of genetically proxied ApoB lowering with CAD risk using the same genetic variants was greater (OR, 0.66 per 1-SD decrease in circulating ApoB [95% CI, 0.63–0.69], *P*=4×10^−73^; ratio of effects, 3.09 [95% CI, 2.29–4.60], *P*<1×10^−6^). Associations were also significant with the MR-Egger method (PAD OR, 0.91 [95% CI, 0.86–0.98], *P*=0.009; CAD OR, 0.70 [95% CI, 0.65–0.76], *P*=8×10^−20^).

Next, we compared the associations between genetically proxied ApoB lowering and ASCVD outcomes in gene-based analyses, identifying potential heterogeneous effects on PAD and CAD risk. We identified protective effects on CAD or PAD for 11 of 12 ApoB target genes (Figure 3B and Table XVI in the Data Supplement). Although several associations did not achieve statistical significance for PAD specifically, associations were generally in the risk-decreasing direction, except for the *HMGCR* locus, which trended toward increased PAD risk (although 95% CIs did not exclude a small protective effect on PAD). Consistent with the overall polygenic analysis, genetically proxied ApoB lowering at the *ABCG5*, *PCSK9*, *APOB*, *LDLR*, and *ANGPTL8* loci had significantly greater protective effects on CAD compared with PAD (ratio of effect estimates >1, FDR <0.05; Figure 3C). Results were similar, although with wider CIs, when we considered the MR-Egger method (Table XVI in the Data Supplement).

**Figure 4.**
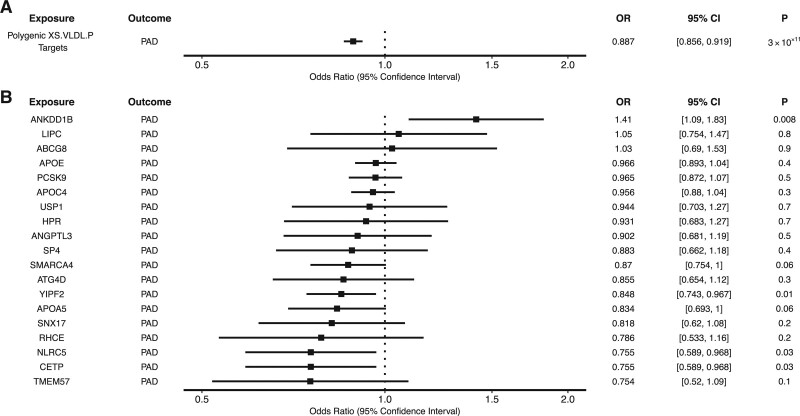
**Associations between extra-small very-low-density lipoprotein particle concentration (XS.VLDL.P)–associated genes and peripheral artery disease (PAD).** Polygenic and gene-specific mendelian randomization (MR) were performed to estimate the association between the apolipoprotein B (ApoB)–lowering effect of XS.VLDL.P–associated genes and PAD. Gene-specific MR examining the impact of ApoB-associated variants in or near (±200 kb) each XS.VLDL.P–associated gene, with polygenic targets denoting the aggregate impact of variants in or near these genes. Odds ratios (ORs) represent the change in disease risk per 1-SD decrease in ApoB.

We also performed polygenic and gene-specific MR analyses to explore whether XS.VLDL.P–associated genes identified in the TWAS analysis were associated with PAD risk. In the polygenic analysis, ApoB lowering proxied by genetic variants located within or near XS.VLDL.P–associated genes was associated with a reduced risk of PAD (OR, 0.89 per 1-SD reduction in ApoB [95% CI, 0.86–0.92]; *P*=3×10^−11^; Figure 4 and Table XVII in the Data Supplement). In gene-specific analyses, ApoB lowering at the *CETP*, *NLRC5*, and *YIPF2* loci were significantly associated with decreased PAD risk, whereas ApoB lowering at the *ANKDD1B* locus was associated with increased PAD risk (Figure 4). CIs were wider with the MR-Egger method (Table XVII in the Data Supplement), although the association remained significant at the polygenic level.

### Circulating Lp(a) and PAD Risk

Because previous studies have identified an association between genetically predicted Lp(a) and CAD,^28,31^ we investigated the relationship between circulating Lp(a) and PAD risk. Unlike the other major lipoprotein-related traits that are genetically correlated and share a complex polygenic architecture, circulating Lp(a) levels are regulated primarily by genetic variants acting specifically at the *LPA* locus on chromosome 6.^32^ Consistent with this architecture, we did not identify significant genetic correlations between Lp(a) and other lipoprotein-related traits (Table XVIII in the Data Supplement). In MR analyses, increases in genetically predicted Lp(a) were associated with increased risk of PAD [OR, 1.04 per 10–mg/dL increase in Lp(a) [95% CI, 1.03–1.04]; *P*=3×10^−32^], and the effect appeared linear across the range of Lp(a) (Table XIX and Figure I in the Data Supplement). This association was not attenuated in multivariable MR accounting for the associations of these genetic variants with ApoB (OR, 1.04 [95% CI, 1.03–1.04]; *P*=1.0×10^−33^). For comparison, genetically predicted Lp(a) was also associated with increased risk of CAD (OR, 1.06 [95% CI, 1.05–1.06]; *P*=1×10^−94^). Genetically predicted Lp(a) was more strongly associated with CAD compared with PAD (ratio of effects, 1.62 [95% CI, 1.35–1.98]; *P*<1×10^−6^).

## Discussion

We integrated several large genetic data sets and an array of statistical genetics, molecular epidemiology, and bioinformatic techniques to uncover novel causal relationships between circulating lipoprotein-related traits and PAD, and we compared these findings with CAD. First, we identified ApoB as the primary major circulating lipoprotein-related trait responsible for risk of PAD, similar to CAD. Next, we prioritized XS.VLDL.P as the ApoB-associated subfraction most strongly associated with PAD risk, in contrast to CAD, for which L.LDL.P was the most strongly associated lipoprotein subfraction. We identified genes involved in the regulation of important ApoB-containing lipoprotein subfractions, which may represent directed targets for novel PAD prevention and treatment strategies. We explored the impact of ApoB lowering on PAD and uncovered the potential for the XS.VLDL.P pathway to be targeted to reduce PAD risk. Last, we identified an association between Lp(a) and PAD that is independent of ApoB.

These results highlight similarities and differences in the roles of circulating lipoproteins for PAD and CAD. Our primary analysis identified ApoB as the major lipoprotein-related trait responsible for both PAD and CAD risk. This finding is consistent with a recent meta-analysis of 22 studies (including 1892 PAD cases and 30 937 controls) that found significantly higher ApoB levels among PAD cases compared with controls.^33^ A nested case-control study within the PHS (Physicians’ Health Study) similarly identified baseline ApoB levels (in addition to several other lipid fractions) as a significant predictor of incident PAD.^34^ In contrast, a large observational study including 31 657 participants of 5 prospective Finnish cohorts did not detect an association between ApoB levels and incident PAD but may have been limited by a low incidence of PAD (498 cases) and by defining PAD on the basis of hospitalization codes.^35^ Similarly, while the WHS (Women’s Health Study) did not identify an association between baseline ApoB and incident PAD,^5^ differences in PAD case definitions, ascertainment, and demographics (incident PAD in WHS versus prevalent in MVP; women in WHS versus predominately men in MVP and PHS) may account for these differences. Our prioritization of ApoB as the most important lipoprotein-related risk factor for CAD is consistent with recent MR studies establishing ApoB as the primary risk factor for CAD.^15,16^ We also identified a modest but significant association between Lp(a) and PAD that was independent of ApoB, consistent with previous MR studies linking Lp(a) to CAD.^28,36^ In the setting of strong epidemiological and genetic correlation between CAD and PAD, it is not surprising that these 2 manifestations of ASCVD share ApoB and Lp(a) as common risk factors. However, we identified a stronger effect of ApoB on CAD than PAD, which has implications for risk stratification and treatment.

ApoB-containing particles exist on a spectrum of varying sizes, densities, and particle compositions, and identification of specific subfractions that contribute to different forms of ASCVD may have implications for pathophysiology, molecular mechanisms, risk stratification, and treatment.^37^ Variability in the distribution of ApoB within lipoprotein subfractions may contribute to differential risk of PAD compared with CAD. Indeed, clinical observations have suggested that type III hyperlipoproteinemia (familial dysbetalipoproteinemia), a disorder specifically associated with VLDL remnant particles, may be a greater risk factor for PAD than for CAD.^37^ In our genetic analyses of ApoB-containing lipoprotein subfractions using MR-BMA, XS.VLDL.P was the primary ApoB-containing subfraction contributing to PAD risk. In contrast, L.LDL.P was the primary ApoB-containing subfraction contributing to CAD risk. These results are consistent with a recent observational study exploring the effect of circulating lipoproteins and metabolites on incident PAD and CAD among 31 657 participants of 5 prospective Finnish cohorts that found a strong association between XS.VLDL.P and incident PAD, with no significant association between L.LDLP and incident PAD.^35^ The primary effect of XS.VLDL.P on PAD implicates an important role for triglyceride-rich lipoproteins and remnant particles in the pathogenesis of PAD. This stands in contrast to the effect of L.LDL.P on CAD, which suggests that LDL-associated lipoprotein fractions may play a more important role in the pathogenesis of CAD.

Epidemiologically, small (rather than large) LDL particles have traditionally been associated with CAD risk, serving as a marker of atherogenic dyslipidemia, particularly in the context of metabolic syndrome.^38^ Various mechanisms have been proposed to explain this relationship, including increased ability of small LDL particles to penetrate the arterial wall and increased susceptibility to oxidation. While a recent systematic review and meta-analysis found that both small dense LDL particles and concentration were associated with risk of CAD across 21 studies representing >30 000 subjects, there was substantial heterogeneity across studies, and observational findings more broadly may be limited by residual environmental confounding.^39^ In contrast, 2 recent observational studies have suggested that remnant cholesterol is more strongly predictive of ASCVD events than other lipoprotein-related traits.^40,41^ The prioritization of L.LDL.P does not preclude the possibility that small LDL or remnant particles also cause ASCVD. Whether small LDL or triglyceride-rich lipoproteins represent causal risk factors rather than a consequence of other metabolic derangements and the specific mechanisms by which different ApoB-containing subfractions contribute to ASCVD risk require further study. A previous MR analysis proposed a mechanism by which remnant particles causally increase inflammation (as measured by C-reactive protein level), whereas no inflammatory effect was detected in the setting of genetically proxied elevations in LDL-C.^42^

Although both PAD and CAD are manifestations of ASCVD, there are pathophysiological differences between the 2 diseases that may provide a basis for targeted treatment strategies.^43^ Observational and genetic studies have suggested that the influence on common cardiovascular risk factors may vary across ASCVD outcomes, for example, with smoking more strongly associated with PAD and blood pressure more strongly associated with CAD, among others.^44–47^ Similarly, although ApoB represents a common lipoprotein risk factor for PAD and CAD, we identified differences in predicted response to ApoB-lowering treatment, underscoring potential differences in the role of circulating lipoproteins on these ASCVD outcomes. We demonstrate that although ApoB lowering is expected to have favorable effects on both PAD and CAD risk, the relative benefit is expected to be significantly greater for CAD risk reduction compared with PAD risk reduction. Although large randomized, controlled trials, genetic studies, and observational evidence have highlighted the importance of LDL-C and ApoB-lowering in reducing ASCVD outcomes overall, these studies have focused primarily on major adverse cardiovascular events and CAD outcomes.^7,48,49^ Our results reveal that the ApoB-lowering effect of several clinically approved and clinical trial-stage drug targets is predicted to differ between PAD and CAD, a finding that may have implications for both drug discovery and treatment paradigms. Although we identified varied effects across ApoB-lowering targets, these results should not at this point be used to guide treatment decisions, and MR and clinical trial estimates of treatment effects may vary.^50^ Although overall these results support guideline recommendations for the use of ApoB-lowering medications to reduce PAD risk, our results also argue for PAD-specific outcomes to be measured in cardiovascular outcomes trials because the absence of a treatment effect for CAD (or a combined end point) may not exclude PAD-specific effects.^1,49^ For example, the CETP (cholesteryl ester transfer protein) inhibitor torcetrapib was associated with a substantial decrease in the incidence of PAD in a large, placebo-controlled phase III clinical trial, although it was overall associated with increased risk of cardiovascular morbidity and mortality.^51^ The ongoing PROMINENT study (Pemafibrate to Reduce Cardiovascular Outcomes by Reducing Triglycerides in Patients With Diabetes) will evaluate the impact of triglyceride-rich lipoprotein lowering on major cardiovascular events with adjudicated PAD events as a secondary end point of the trial.^52^

Last, our polygenic and gene-based drug target analyses highlighted potential targets for directed treatment strategies. Because lipid-lowering medications induce specific changes in the circulating lipoprotein profile, our results suggest that specific drug target identification may play an important role in identifying PAD-focused treatments.^53^ For example, genes associated with circulating XS.VLDL.P were enriched in pathways related to triglyceride-rich lipoprotein metabolism. Although our polygenic and gene-specific MR analyses suggested that both currently available and proposed ApoB-lowering therapies would be expected to reduce PAD risk, we used MR to further highlight the XS.VLDL.P pathway as a potentially novel therapeutic target. Whether these genes and pathways represent pharmacological targets that ultimately affect PAD outcomes warrants further study.

### Limitations

This study should be interpreted within the context of its limitations. First, this study focused on prevalent PAD outcomes ascertained from electronic health records. The effect of lipoprotein-related traits may vary across specific incident PAD outcomes, including intermittent claudication, rest pain, tissue loss, and amputation. Second, PAD outcomes were studied among primarily male participants of the Veterans Affairs MVP, and although participants were of diverse ancestries, further studies among other populations are warranted to improve the generalizability of these findings. Third, MR effect estimates reflect lifelong genetic exposures and may not accurately reflect the magnitude of benefit of shorter-term pharmacological interventions.^54^ Similarly, although we did not detect pleiotropic effects in MR-Egger analyses, we cannot exclude the possibility that genetic variants located within targets of lipid-lowering therapies may influence other cardiometabolic traits. Thus, our drug target MR findings should not be used to guide clinical decisions on lipid-lowering therapies at this stage. Fourth, when correlated exposures exist within a common pathway, the MR-BMA method identifies the most proximate risk factor to the outcome. Circulating levels of each lipid fraction are composed of several subcomponents (eg, ApoB, triglycerides, and cholesterol are found in VLDL, intermediate-density lipoprotein, and LDL compartments and subfractions). Prioritization of ApoB over LDL-C for PAD and CAD risk does not nullify LDL-C as a causal risk factor but indicates that the effects of lipid-lowering therapies are likely to be proportional to the change in ApoB rather than LDL-C. Detailed MR analysis of other lipid subfractions may prioritize additional PAD risk factors and therapeutic targets. Last, larger population-scale GWASs of circulating lipoprotein-related traits and subfractions may enable the development of more robust genetic instruments, which may provide additional insights into the relationships between circulating lipoprotein-related traits and ASCVD.

Overall, this analysis of large genetic data sets identified ApoB as the primary causal lipoprotein-related risk factor for PAD. Diverse effects of ApoB-lowering drug targets and ApoB-containing lipoprotein subfractions on PAD compared with CAD suggest possible biological differences in the pathogenesis of these diseases, with gene expression analyses revealing potential targets for novel PAD therapies.

## Acknowledgments

The authors thank the participants of the VA Million Veterans Program, UK Biobank, CARDIoGRAMplusC4D, and NMR metabolomics studies.

## Sources of Funding

This research is based on data from the MVP, Office of Research and Development, Veterans Health Administration and was supported by award MVP003/MVP028 (I01-BX003362). A list of MVP collaborators is included in the Data Supplement. This work was supported by US Department of Veterans Affairs grant IK2-CX001780 (Dr Damrauer). This publication does not represent the views of the Department of Veterans Affairs or the US government. Dr Zuber is supported by UK Dementia Research Institute at Imperial College London, which is funded by the Medical Research Council, Alzheimer’s Society, and Alzheimer’s Research UK (MC_PC_17114). Dr Aday is supported by NIH K23 HL151871. Dr Gill is supported by the British Heart Foundation Center of Research Excellence (RE/18/4/34215) at Imperial College London and a National Institute for Health Research Clinical Lectureship at St. George’s, University of London (CL-2020-16-001). Dr Burgess is supported by a Sir Henry Dale Fellowship jointly funded by the Wellcome Trust and the Royal Society (grant 204623/Z/16/Z).

## Disclosures

Dr Damrauer receives research support to his institution from RenalytixAI and reports consulting fees from Calico Labs, all outside the current work. Dr Gill is employed part-time by Novo Nordisk. Dr Rader serves on scientific advisory boards for Alnylam, Novartis, Pfizer, and Verve and is a cofounder of Staten Biotechnology. Dr Aday reports consulting fees from OptumCare outside the current work. The other authors report no conflicts.

## Supplemental Material

Expanded Methods and Materials

Data Supplement Figure I

Data Supplement Excel File I (Data Supplement Tables I–XIX)

References 55–60

## Supplementary Material


