# Supplementary material for: Prioritizing the Role of Major Lipoproteins and Subfractions as Risk Factors for Peripheral Artery Disease
Source: Circulation. Author manuscript; Available in PMC 2021 Aug 4. (PMC8323712; doi:10.1161/CIRCULATIONAHA.121.053797)
Supplement: Supplemental Methods and Figures [file EMS128610-supplement-Supplemental_Methods_and_Figures.pdf]

SUPPLEMENTAL MATERIAL

Prioritizing the Role of Major Lipoproteins and Subfractions as Risk Factors for Peripheral Artery Disease

Table of Contents

SUPPLEMENTAL METHODS 2
Mendelian Randomization Bayesian Model Averaging (MR-BMA) 2
UK BioBank Loss-of-Function Burden Analysis 3
SUPPLEMENTAL FIGURES 4
Supplemental Figure I: Association of Lp(a) with PAD and CAD 4
SUPPLEMENTAL TABLES 5

SUPPLEMENTAL METHODS

Mendelian Randomization Bayesian Model Averaging (MR-BMA)

We performed a variable selection method in a multivariable MR framework to prioritize the causal lipoprotein determinants of the outcomes. Multivariable MR extends the basic MR framework to include multiple exposures in one joint model, which is particularly relevant when considering highly correlated traits like blood lipoprotein-related traits as exposures.[22,23] In order to rank and select the likely causal lipoprotein risk factors for PAD, we employed an extension of multivariable MR called Mendelian randomization Bayesian model averaging (MR-BMA), a Bayesian approach for prioritizing causal exposures in a two-sample multivariable MR setting.[14] MR-BMA performs variable selection by evaluating models with all possible combinations of lipoprotein-related traits as exposures and computing the posterior probability for each model that the model contains the true causal risk factors. Unlike other univariate or multivariable MR methods, MR-BMA aims to identify true causal risk factors among correlated traits, rather than estimate the magnitude of effect.[14] The evidential support for each exposure (the marginal inclusion probability) is derived from the sum of all posterior probabilities of the models where the specific exposure was included. We removed influential variants based on the Cook's distance and outliers based on the q-statistic as previously recommended.[14] An empirical permutation procedure was used to calculate p-values which are adjusted for multiple testing using the Benjamini-Hochberg false discovery rate (FDR) procedure, as previously described.[55] Briefly, the expected marginal inclusion probability distribution for each risk factor under the null hypothesis was generated by performing 1,000 permutations of the MR-BMA analysis, holding the SNP-risk factor associations constant and randomly permuting the SNP-outcome associations. The observed marginal inclusion probabilities for each risk factor were then compared to the expected distribution, with p-values computed by $p_j = (r_j +1)/(n_{perm} + 1)$, where r_j represents the rank of the observed marginal inclusion probability of a given risk factor (j) across all permutations ($n_{perm} = 1000$).

UK BioBank Loss-of-Function Burden Analysis

To estimate the effect of damaging mutations in XS.VLDL.P-associated genes on risk of PAD, we performed a burden test among UK Biobank participants who underwent whole exome sequencing (WES). PAD was defined using ICD10 codes from death records and hospital episode stays (HES), ICD9 codes and OPCS4 codes, as previously described.[10] All individuals with >1 code were assigned a case status, whereas all other individuals were assigned a control status. In the complete UK Biobank dataset (N=502,336), we identified 6,329 unique PAD cases (1.26% cases in population). In the UK Biobank WES dataset (N=200,644), we identified 2,147 unique PAD cases (1.07% cases in population). We excluded from this dataset: i) individuals from non-British White ancestry and ii) individuals with excess heterozygosity. For burden analysis, we also excluded related individuals (up to 2nd degree; KING cutoff 0.0884) using PRIMUS while retaining cases preferentially to controls (high_btrait option in PRIMUS)[56]. Our final dataset consisted of 1,668 cases and 152,916 controls (1.08% cases in population). For the 31 selected genes, we selected rare variants (MAF < 0.01) that are either predicted to be damaging (REVEL[57] score > 0.5) or predicted to exert a high-confidence loss-of-function effect using the LoFTEE[58] plugin in VEP[59]. We pooled those variants in a combined burden analysis using the CMC unidirectional burden test implemented in the rvtests software.[25,60] The CMC test was performed using age at baseline, sex and 10 PCs as covariates.

SUPPLEMENTAL FIGURES

Supplemental Figure I: Association of Lp(a) with PAD and CAD

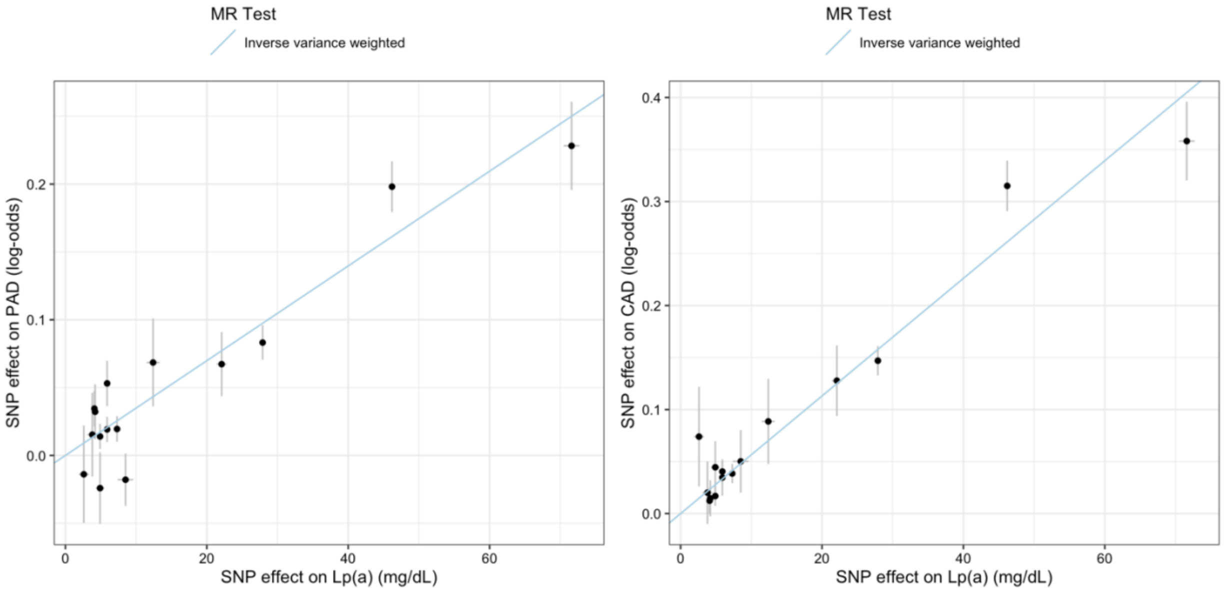

A genetic instrument for Lp(a) was constructed using 15 conditionally-independent genetic variants associated with circulating Lp(a) at the genome-wide significance threshold ($p < 5 \times 10^{-8}$). Plots demonstrate the association between Lp(a) and log-odds of either PAD or CAD. The inverse variance-weighted estimated effect is highlighted.

SUPPLEMENTAL TABLES

Supplemental Table	Sheet	Description
Supplemental Table I	ST1	Main analysis (UK Biobank): Models (i.e. all possible combinations of exposures) ranked by the model posterior probability. Instrumental variables are n=130 independent genetic variants associated with blood lipids in the Global Lipid Genetics Consortium after removing 15 outliers and influential genetic variants. Causal effects are Bayesian estimates for the direct effect (log odds ratios for peripheral artery disease per 1 standard deviation increase in the exposure) and their respective standard errors.
Supplemental Table II	ST2	Main analysis (UK Biobank) before sensitivity analysis: Bayesian multivariable Mendelian randomization (MR-BMA) is used to rank A) most likely causal exposures for peripheral artery disease by marginal inclusion probabilities and B) most likely models (i.e. all possible combinations of exposures) by posterior probabilities. Causal effect estimates represent direct effects of the exposure on the outcome (log odds ratios for peripheral artery disease per 1 standard deviation increase in the exposure), i.e. are A) model-averaged causal effects and B) Bayesian effect estimates for a particular model and their respective standard error. Instrumental variables are all n=145 independent genetic variants associated with blood lipids in the Global Lipid Genetics Consortium.
Supplemental Table III	ST3	Sensitivity analysis for main analysis: A) Influential genetic variants identified by Cook's distance in the top models (M1, M2, ...) and B) Outlying genetic variants identified by the q-statistic in the top models (M1, M2, ...). The multiple testing corrected threshold is q>12.78.
Supplemental Table IV	ST4	Replication analysis (NMR metabolite GWAS): Models (i.e. all possible combinations of exposures) ranked by the model posterior probability. Instrumental variables are n=132 independent genetic variants associated with blood lipids in the Global Lipid Genetics Consortium after removing 13 outliers and influential genetic variants. Causal effects are Bayesian estimates for the direct effect (log odds ratios for peripheral artery disease per 1 standard deviation increase in the exposure) and their respective standard errors.
Supplemental Table V	ST5	Replication analysis (NMR metabolite GWAS) before sensitivity analysis: Bayesian multivariable Mendelian randomization (MR-BMA) is used to rank A) most likely causal exposures for peripheral artery disease by marginal inclusion probabilities and B) most likely models (i.e. all possible combinations of exposures) by posterior probabilities. Causal effect estimates represent direct effects of the

		exposure on the outcome (log odds ratios for peripheral artery disease per 1 standard deviation increase in the exposure), i.e. are A) model-averaged causal effects and B) Bayesian effect estimates for a particular model and their respective standard error. Instrumental variables are all n=145 independent genetic variants associated with blood lipids in the Global Lipid Genetics Consortium.
Supplemental Table VI	ST6	Sensitivity analysis for replication analysis: A) Influential genetic variants identified by Cook's distance in the top models (M1, M2, ...) and B) Outlying genetic variants identified by the q-statistic in the top models (M1, M2, ...). The multiple testing corrected threshold is q>12.66.
Supplemental Table VII	ST7	Coronary artery disease as outcome and 5 lipid measurement from UK Biobank as exposures: Bayesian multivariable Mendelian randomization (MR-BMA) is used to rank A) most likely causal exposures for coronary artery disease by marginal inclusion probabilities and B) most likely models (i.e. all possible combinations of exposures) by posterior probabilities. Causal effect estimates represent direct effects of the exposure on the outcome (log odds ratios for coronary artery disease per 1 standard deviation increase in the exposure), i.e. are A) model-averaged causal effects and B) Bayesian effect estimates for a particular model and their respective standard error. Instrumental variables are all n=136 independent genetic variants associated with blood lipids in the Global Lipid Genetics Consortium after excluding 11 outliers.
Supplemental Table VIII	ST8	Coronary artery disease as outcome and 5 lipid measurement from the NMR metabolite GWAS as exposures: Bayesian multivariable Mendelian randomization (MR-BMA) is used to rank A) most likely causal exposures for coronary artery disease by marginal inclusion probabilities and B) most likely models (i.e. all possible combinations of exposures) by posterior probabilities. Causal effect estimates represent direct effects of the exposure on the outcome (log odds ratios for coronary artery disease per 1 standard deviation increase in the exposure), i.e. are A) model-averaged causal effects and B) Bayesian effect estimates for a particular model and their respective standard error. Instrumental variables are all n=135 independent genetic variants associated with blood lipids in the Global Lipid Genetics Consortium after excluding 11 outliers.
Supplemental Table IX	ST9	Multivariable subfraction analysis for peripheral artery disease: Top 10 models (i.e. all possible combinations of exposures) ranked by the model posterior probability. Genetic associations with particle concentrations of apolipoprotein B-containing lipid subfractions

		from an NMR metabolite genome-wide association study were used as exposures and genetic associations with peripheral artery disease (transethnic analysis) were used as the outcome. Instrumental variables are n=144 independent genetic variants associated with apolipoprotein B in UK Biobank after removing 10 outliers and influential genetic variants. Causal effects are Bayesian estimates for the direct effect (log odds ratios for peripheral artery disease per 1 standard deviation increase in the exposure) and their respective standard errors.
Supplemental Table X	ST10	Multivariable subfraction analysis for coronary artery disease: Top 10 models (i.e. all possible combinations of exposures) ranked by the model posterior probability. Genetic associations with particle concentrations of apolipoprotein B-containing lipid subfractions from an NMR metabolite genome-wide association study were used as exposures and genetic associations with coronary artery disease (CARDIoGRAMplusC4D 1000 Genomes GWAS) were used as the outcome. Instrumental variables are n=144 independent genetic variants associated with apolipoprotein B in UK Biobank after removing 10 outliers and influential genetic variants. Causal effects are Bayesian estimates for the direct effect (log odds ratios for peripheral artery disease per 1 standard deviation increase in the exposure) and their respective standard errors.
Supplemental Table XI	ST11	TWAS results for XS.VLDL.P
Supplemental Table XII	ST12	TWAS results for L.LDL.P
Supplemental Table XIII	ST13	Results of Gene Ontology (GO) Biological Processes enrichment for genes associated with either XS.VLDL.P or L.LDL.P
Supplemental Table XIV	ST14	Results of Gene Ontology (GO) Biological Processes enrichment for genes associated with XS.VLDL.P
Supplemental Table XV	ST15	Results of loss-of-function burden analysis testing for association between damaging mutations in XS.VLDL.P-associated genes and PAD among UK Biobank participants
Supplemental Table XVI	ST16	Results of gene-level ApoB pathway MR. Exposure based on genetic variants associated with ApoB located with genes encoding protein targets of ApoB-lowering therapies
Supplemental Table XVII	ST17	Results of gene-level ApoB pathway MR. Exposure based on genetic variants associated with ApoB located with genes encoding protein targets of XS.VLDL.P-assocatied genes
Supplemental Table XVIII	ST18	Genetic correlation among lipids as determined by cross-trait LD-score regression
Supplemental Table XIX	ST19	Genetic instruments for Lipoprotein(a) (mg/dL) and corresponding SNP effects for PAD and CAD
